# The association of 5HT2A and 5HTTLPR polymorphisms with Alzheimer’s disease susceptibility: a meta-analysis with 6945 subjects

**DOI:** 10.18632/oncotarget.23611

**Published:** 2017-12-22

**Authors:** Liang Tang, Jianming Li, Huaiqing Luo, Meihua Bao, Ju Xiang, Yiwei Chen, Yan Wang

**Affiliations:** ^1^ Department of Human Anatomy, Histology and Embryology, Institute of Neuroscience, Changsha Medical University, Changsha, PR China; ^2^ Department of Neurology, Xiang-Ya Hospital, Central South University, Changsha City, Hunan Province, PR China; ^3^ School of Basic Medical Science, Changsha Medical University, Changsha, PR China; ^4^ Experiment Center for Function, Changsha Medical University, Changsha, PR China

**Keywords:** Alzheimer’s disease (AD), 5-hydroxytryptophan 2A Receptor (5HT2A), serotonin transporter (5HTT), Apolipoprotein E (APOE), meta-analysis

## Abstract

Alzheimer’s disease (AD) is a progressive neurodegenerative disease. Relationships of 5HT2A and 5HTTLPR polymorphisms and AD risk have been widely investigated previously, whereas results derived from these studies were inconclusive and controversial. The aim of this study was to investigate the association of the 5-HT2A and 5HTTLPR polymorphisms and AD using a meta-analysis of existing literatures. Studies were collected using PubMed, Web of Science, the Cochrane Library databases, Chinese National Knowledge Infrastructure (CNKI) and Embase. Pooled odds ratios (ORs) with 95% confidence intervals (CIs) were used to assess associations. As a result, a total of 7 publications for 5-HT2A T102C and 16 publications for 5HTTLPR (L/S) comprised 3255 cases and 3690 controls fulfilled the inclusion criteria. Significant association was covered between allelic and recessive models of 5-HT2A T102C and AD (allelic model: *p* = 0.003, OR [95% CI] = 1.23 [1.07, 1.40]; recessive model: *p* = 0.03, OR [95% CI] = 1.28 [1.02, 1.59]). Subsequently, we conducted subgroup analysis for 5-HT2A T102C polymorphism based on ethnicities and APOE ε4, and identified a significantly increased risk for the allelic and dominant models of 5-HT2A T102C and AD in Asian subgroup (allelic model: *p* = 0.002, OR [95% CI] = 1.42 [1.14, 1.78]; dominant model: *p* = 0.02, OR [95% CI] = 1.60 [1.09, 2.35]) and subgroup without APOE ε4 (allelic model: *p* = 0.02, OR [95% CI] = 1.44 [1.05, 1.99]; dominant model: *p* = 0.0008, OR [95% CI] = 2.49 [1.46, 4.25]). Nevertheless, the pooled analyses suggested no significant association between allelic, dominant, and recessive models of 5HTTLPR (L/S) and AD (*p* > 0.05). In conclusion, our meta-analysis demonstrates that 5HT2A C10T, but not 5HTTLPR (L/S), might increase risk for AD.

## INTRODUCTION

Alzheimer’s disease (AD), characterized by progressive memory and language impairment, cognitive deficits, and other behavioral and psychological symptoms of dementia (BPSD), is a progressive neurodegenerative disorder [1–2]. The mechanism of AD is complex and not well known yet. Apart from environment factors such as education level and life style, genetic contribution may partly determinate the risk of AD [3–5]. The well-studied genetic risk factor for AD should be the apolipoprotein E (APOE) ε4 allele. It was suggested to be a susceptible factor to both familial and sporadic AD [6–8]. However, this variant accounts to only part of genetic susceptibility to AD [9]. Therefore, further gene polymorphisms may confer additional risk to develop AD.

Serotonin (5-hydroxytryptamine, 5-HT) is a key neurotransmitter involved in many aspects of psychological processes including mood, aggression, impulsivity, and anxiety in human and animal [10–13]. Serotonin dysfunction has been implicated in many psychiatric diseases including AD [14]. The action of 5-HT is mediated by 5HT receptors. Multiple 5-HT receptors have been identified. Increasing evidences suggest that 5-HT receptors especially 5HT2A and 5HT1A have impartment role in the development of AD [15–17]. In addition, large number of neurobiological researches have suggested a decrease in density and specific binding of the 5HT2A receptor in AD patients’ brain [18–19]. Following 5-HT release, the serotonin reuptake transporter (5-HTT) is thought to be the principal regulation site of the serotonin levels by facilitating reuptake of 5-HT from the synaptic cleft to its receptors in the central nervous system [20]. The 5-HTT may therefore be also involved in the pathogenesis of AD.

Polymorphisms in the serotonin-related genes were demonstrated to be associated with the risk of AD in recent studies. The most commonly and widely studied polymorphisms should be the 5-HT2A (C102T) and SLC6A4 (5HTTLPR) [21–23]. The 5HT2A C102T is a variant change in exon 1 that does not alter the serine at position 34 and was shown to contribute to lower transcriptional activity than the 5HT2A 102C [24]. Increasing case-control studies have investigated the association of 5-HT2A C102T and AD and reported conflict results. While, most of the studies revealed negative results [21, 25–26]. As for 5-HTTLPR, an insertion or deletion of a 44-bp fragment in the promoter region of 5-HTT gene (SLC6A4), was found to regulate 5-HTT promoter activity by cAMP and protein kinase C [27, 29]. The short (S) allele (deletion) is associated with a lower rate of 5HTT transcription than the long (L) allele (insertion) and therefore may reduce 5HT reuptake capacity and lead to alterations in serotonergic neurotransmission [28, 30]. The genetic correlation of 5-HTTLPR (L/S) and AD was firstly identified by Li et al. in British population [31]. However, this positive result can only be replicated in several Caucasian populations [32–33], but not in Asian populations [34–36]. These discrepancies may be due to insufficient calculated power, different ethnicities, and limited sample sizes in individual studies.

In light of these controversial and inconclusive observations, we conducted a meta-analysis to investigate the possible role of 5-HT2A (C102T) and 5HTTLPR (L/S) polymorphisms in susceptibility of AD.

## RESULTS

### Characteristics of the published studies

**Figure 1:**
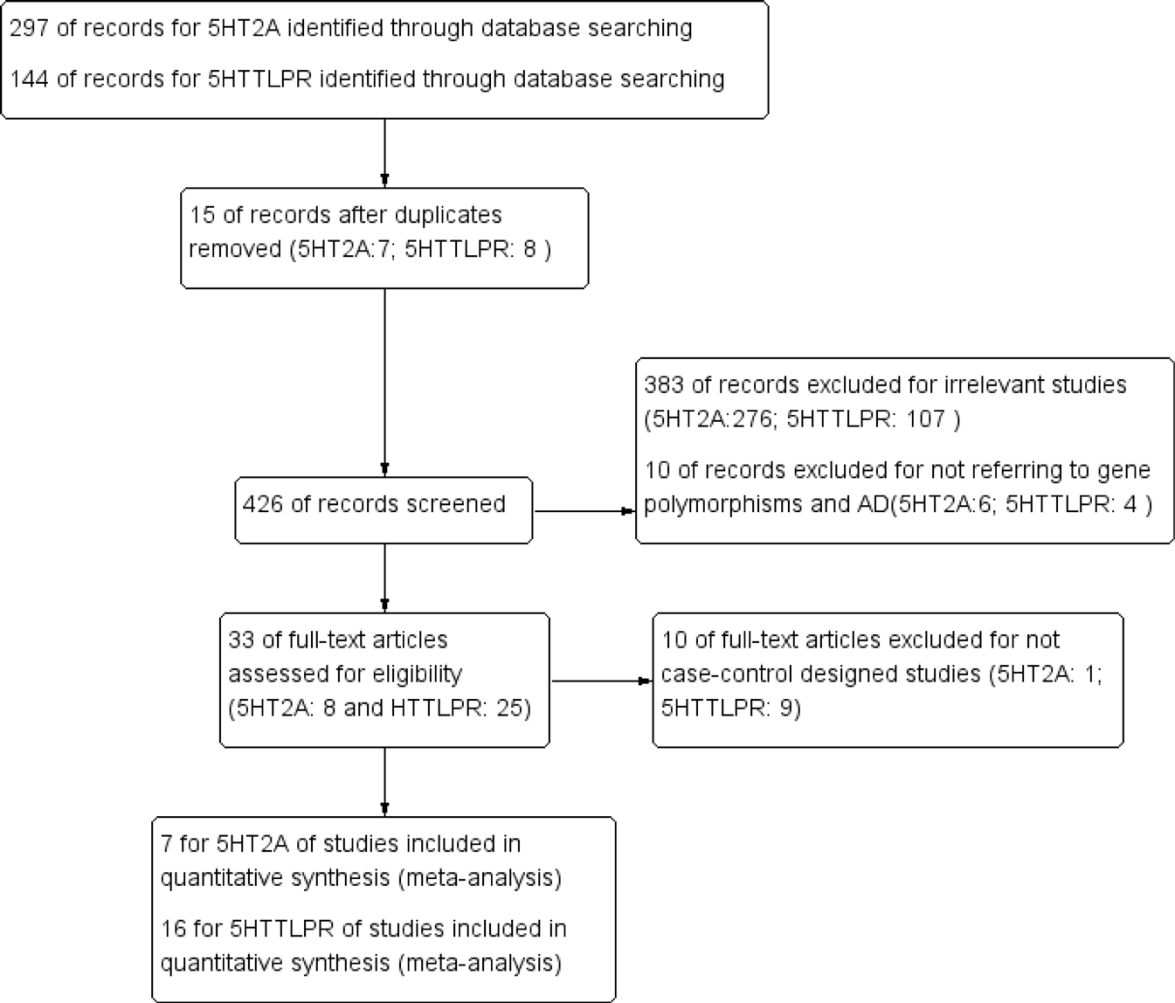
PRISMA flow chart of studies inclusion and exclusion

As shown in Figure 1, we initially retrieved 441 articles (297 for 5HT2A and 144 for 5HTTLPR) from databases. After screening the titles, abstracts, and full text, 15 were excluded for duplicated studies (7 for 5HT2A and 8 for 5HTTLPR). 383 were excluded for irrelevant studies (276 for 5HT2A and 107 for 5HTTLPR). 10 were excluded for not referring to the genetic association of 5-HT2A T102C and 5HTTLPR (L/S) and AD (6 for 5-HT2A T102C and 4 for 5HTTLPR (L/S)). 10 were excluded for not case-control designed studies (1 for 5HT2A and 9 for 5HTTLPR). Finally, a total of 7 articles for 5HT2A C102T [22, 25–26, 37–40] and 16 articles for 5HTTLPR (L/S) [1, 22–23, 31–36, 40–46] involving 3255 cases and 3690 controls were recruited in the present meta-analysis. For 5HT2A C102T, there were 4 studies referring to Caucasians [22, 26, 37, and 40] and 3 studies referring to Asians [25, 38–39]. In addition, 2 studies reported APOE ε4 (with/without) subtypes of AD cases and controls [38–39]. As for 5HTTLPR (L/S), there were 12 studies referring to Caucasians [1, 22–23, 31–33, 40, 42–46] and 4 studies referring to Asians [34–36, 41]. In addition, 5 studies reported APOE ε4 (with/without) subtypes of AD [33, 35–36, 42, 44]. The genetic distributions of the control group in individual study were consistent with the Hardy-Weinberg equilibrium (HWE). The Newcastle-Ottawa Scale (NOS) [47] was used for quality assessment. And all of the studies achieved moderately high quality scores above 7 (Table 1, Supplementary Table 1).

**Table 1:** Characteristics of eligible studies included in the meta-analysis

Gene	Author (year)	Ethnicity	Number of cases	Number of controls	Age(case/control)	M/F(case: control)	source	result	HWE	Quality Assessment (NOS)
5HT2A C102T	Lam et al. 2004	Chinese	87	75	77.4 ± 6.6/73.9 ± 5.6	NA	HB	> 0.05	> 0.05	7
	Rocchi et al. 2003	Itailan	135	90	72.4 ± 7.8/70.2 ± 9.1	45/90:30/60	HB	> 0.05	> 0.05	9
	Micheli et al. 2006	Itailan	208	116	71.8 ± 9.5/70.8 ± 4.6	76/132:58/58	PB	> 0.05	> 0.05	9
	Nacmias et al. 2001	Itailan	83	72	65.4 ± 8.4/74.5 ± 25.1	NA	HB	> 0.05	> 0.05	8
	Ueno et al. 2007	Japanese	164	164	73.1 ± 8.3/73.0 ± 9.5	52/112:68/96	HB	<0.05	> 0.05	9
	Zhang et al. 1999	Chinese	82	97	75 ± 8/70 ± 7	NA	HB	> 0.05	> 0.05	7
	Fehér et al.2013	Hungarian	252	234	75.2 ± 7.4/74.6 ± 6.9	118/134:110/124	HB	<0.05	> 0.05	9
5HTTLPR L/S	Kunugi et al. 2000	Japanese	123	326	79 ± 6/57 ± 8	35/88:151/185	NA	> 0.05	> 0.05	7
	Ha et al 2004	Korean	65	43	74.9 ± 6.9/73.1 ± 3.8	27/38:20/41	HB	> 0.05	> 0.05	9
	Tsai et al.2001	Chinese	136	175	72.6 ± 5.3/71.5 ± 6.4	76/60:75/102	HB	> 0.05	> 0.05	9
	Ueki et al. 2007	Japanese	200	200	73.3 ± 7.9/ 72.6 ± 8.8	66/134:7/127	HB	> 0.05	> 0.05	9
	Fehér et al.2013	Hungarian	252	234	75.2 ± 7.4/74.6 ± 6.9	118/134:110/124	HB	> 0.05	> 0.05	9
	Lorenzi et al. 2010	Itailan	218	54	75.49 ± 8.28/66.79 ± 6.99	77/141:28/26	HB	<0.05	> 0.05	9
	Forero et al. 2006	Colombian	106	97	73.3 ± 8.8/72.2 ± 8.7	NA	HB	> 0.05	> 0.05	7
	Gru¨nblatt et al. 2009	Austrian	127	479	NA	49/78:198/281	HB	> 0.05	> 0.05	9
	Hu et al. 2000	Germany	50	99	NA	NA	HB	> 0.05	> 0.05	7
	Li et al. 1997	British	196	257	82.5 ± 6.7/70.4 ± 8.5	NA	HB	<0.05	> 0.05	8
	Oliveira et al. 1998	Brazil	81	244	70.02 ± 8.13/75.6 ± 10.2	NA	NA	<0.05	> 0.05	7
	Polito et al. 2011	Itailan	235	207	78.6 ± 9.8/ 77.0 ± 9.3	74:161:69:138	HB	<0.05	> 0.05	9
	Seripa et al. 2008	Itailan	105	114	78.42 ± 7.46/78.42 ± 7.46	34/71:69/45	PB	> 0.05	> 0.05	9
	Sukonick et al. 2001	American	58	79	79.0 ± 8.0/73.1 ± 8.0	26/32:29/50	HB	<0.05	> 0.05	9
	Zill et al. 2000	Germany	84	118	73 ± 9/47 ± 12	36/48:55/63	NA	> 0.05	> 0.05	8
	Micheli et al. 2006	Itailan	208	116	71.8 ± 9.5/70.8 ± 4.6	76/132:58/58	PB	> 0.05	> 0.05	9

Abbreviations: 5HT2A: 5-hydroxytryptophan 2A Receptor; 5HTTLPR: 5HTT gene-linked polymorphic region; L: long; S:short; M: male; F; female; HB: hospital based; PB: population based; HWE: Hardy-Weinberg equilibrium; NOS: Newcastle-Ottawa Scale.

### Meta-analysis: 5HT2A (C102T) and Alzheimer’s disease

**Figure 2:**
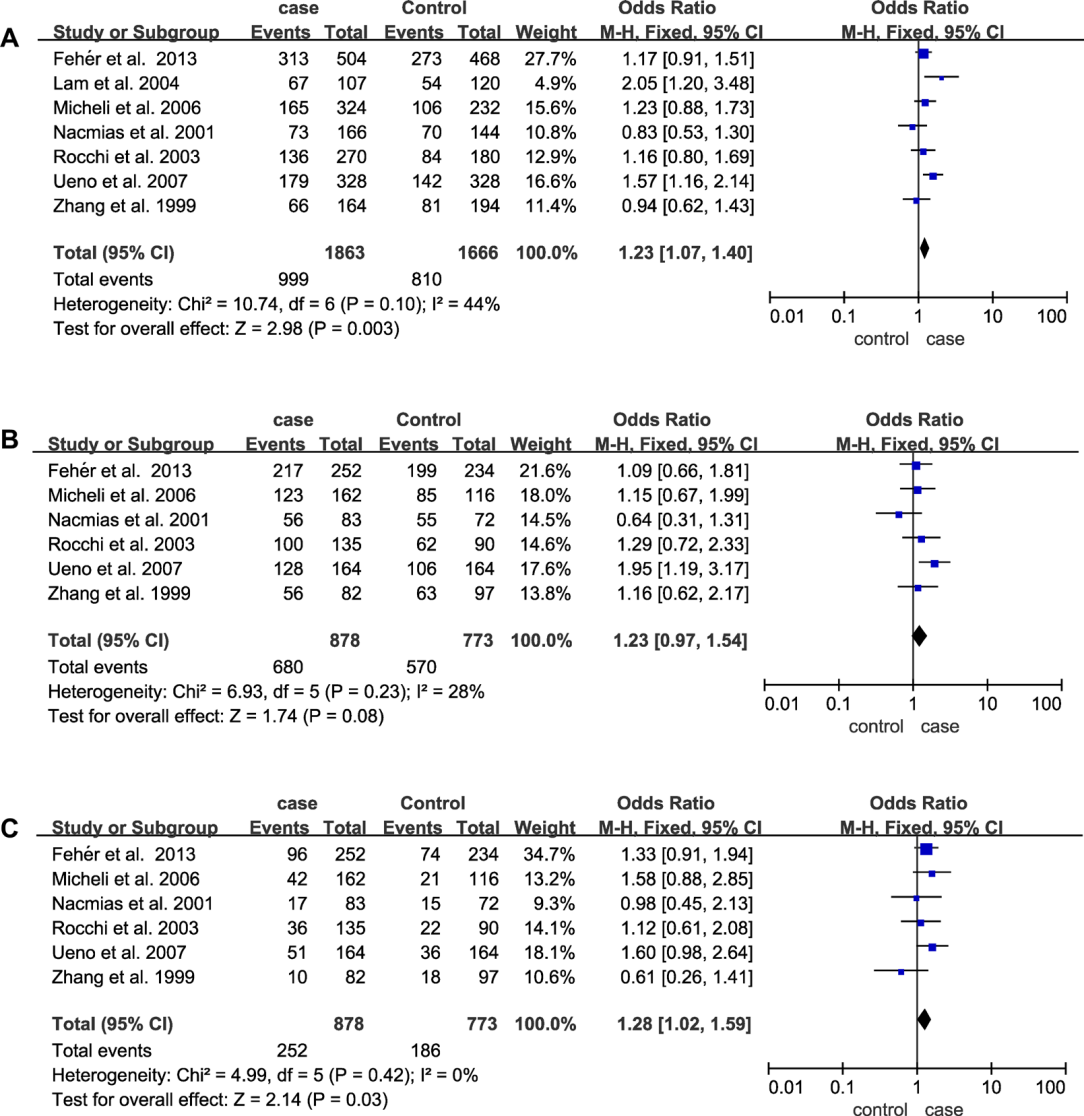
Forest plots of odds ratios for the association between 5HT2A C102T and AD (**A**) Allelic model; (**B**) Dominant model; (**C**) Recessive model.

The main results of the meta-analysis of the association between 5HT2A (C102T) and AD are listed in Table 2. A total of 7 articles including 1011 cases and 848 controls were recruited. Increased AD risk could be shown in both the allelic (OR = 1.23; 95% CI = 1.07–1.40) and recessive models (OR = 1.28; 95% CI = 1.02–1.59), but not in dominant model (*p* = 0.08) of 5HT2A C102T (Figure 2). Subgroups analysis based on ethnicities showed a significant association between allelic and dominant models of 5HT2A (C102T) and AD in Asian subgroup (allelic model: OR = 1.42; 95% CI = 1.14–1.78; dominant model: OR = 1.60; 95% CI = 1.09–2.35). In addition, subgroup analysis stratified by APOE ε4 revealed that the distributions of allelic contrast (OR = 1.44; 95% CI = 1.05–1.99) and dominant model (OR = 2.49; 95% CI = 1.46–4.25) of 5HT2A C102T were significantly increased in AD subgroup without APOE ε4, but not in AD subgroup with APOE ε4 (*p* > 0.05) (Table 2).

**Table 2:** The association between 5HT2A C102T and Alzheimer’s disease

SNPs (minor allele)	Genetic Model	Number of studies	Numbers		Test of association		Model	Test of heterogeneity	
			case	control	OR[95% CI]	*p*-Value		*P* value	I^2^ (%)
5HT2A (C)	Allelic(C)								
	total	7	1863	1666	1.23 [1.07, 1.40]	0.003	F	0.10	44
	Asian	3	599	642	1.42 [1.14, 1.78]	0.002	R	0.05	66
	Caucasian	4	1264	1024	1.13 [0.95, 1.33]	0.16	F	0.54	0
	With APOE ε4	2	256	102	0.98 [0.28, 3.38]	0.94	R	0.01	84
	Without APOE ε4	2	236	420	1.44 [1.05, 1.99]	0.02	F	0.75	0
	Dominant(CC+CT/TT)								
	total	6	878	773	1.23 [0.97, 1.54]	0.08	F	0.23	28
	Asian	2	246	261	1.60 [1.09, 2.35]	0.02	F	0.20	38
	Caucasian	4	632	512	1.05 [0.79, 1.40]	0.72	F	0.49	0
	With APOE ε4	2	128	51	0.73 [0.35, 1.52]	0.39	F	0.17	48
	Without APOE ε4	2	118	210	2.49 [1.46, 4.25]	0.0008	F	0.79	0
	Recessive(CC/CT+TT)								
	total	6	878	773	1.28 [1.02, 1.59]	0.03	F	0.42	0
	Asian	2	246	261	1.24 [0.81, 1.88]	0.32	R	0.05	74
	Caucasian	4	632	512	1.29 [0.99, 1.68]	0.06	F	0.76	0
	With APOE ε4	2	128	51	1.84 [0.11, 29.95]	0.67	R	0.009	85
	Without APOE ε4	2	118	210	1.01 [0.59, 1.73]	0.98	F	0.42	0

Abbreviations: 5HT2A: 5-hydroxytryptophan 2A Receptor; APOE; Apolipoprotein E; R: random model; F: fixed model; OR: odds ratios; CIs: confidence intervals.

### Meta-analysis: 5HTTLPR (L/S) and Alzheimer’s disease

**Figure 3:**
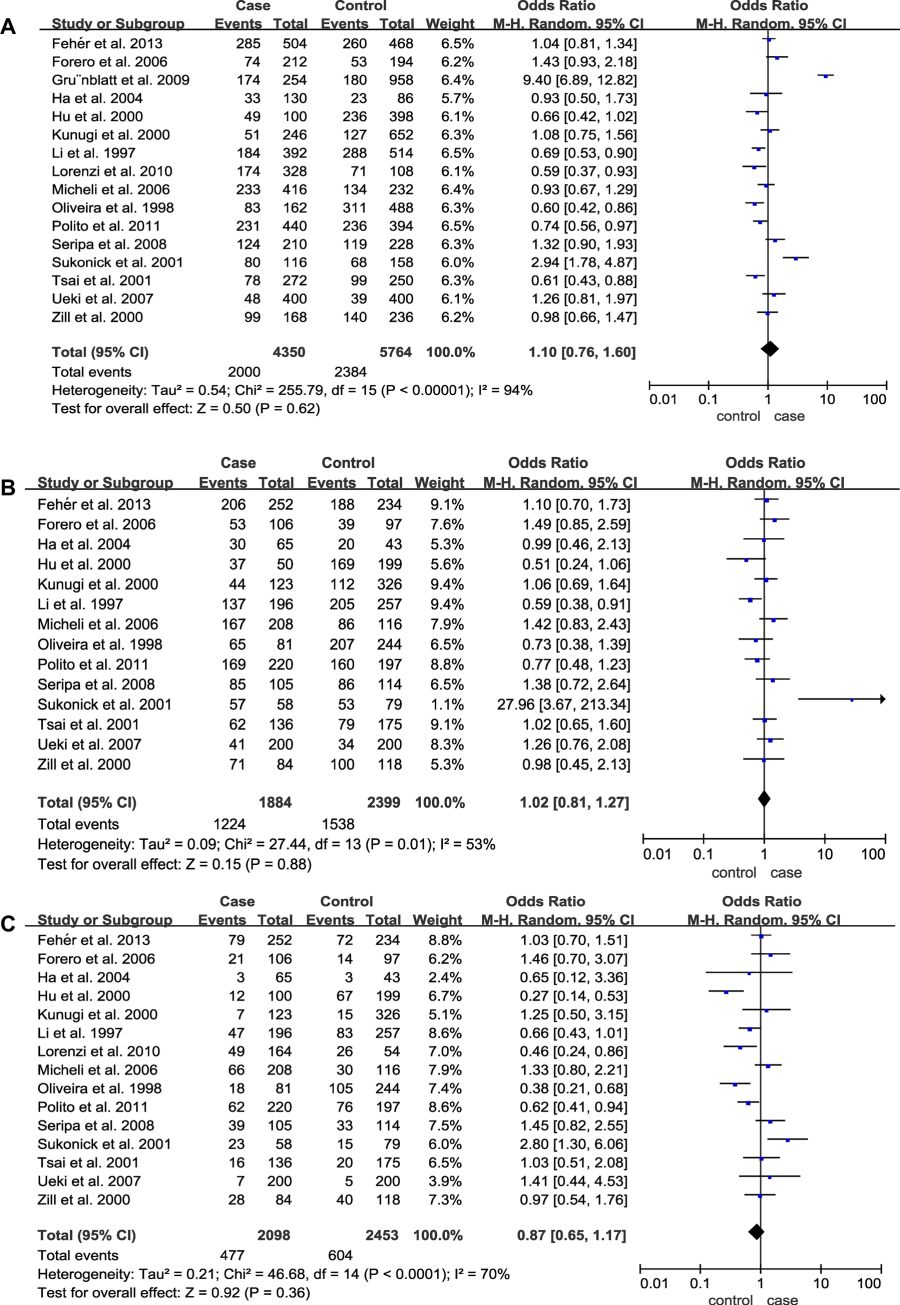
Forest plots of odds ratios for the association between 5HTTLPR (L/S) and AD (**A**) Allelic model; (**B**) Dominant model; (**C**) Recessive model.

For 5HTTLPR (L/S), a total of 16 case-control studies containing 2244 cases and 2842 controls were involved. Nevertheless, the pooled ORs for the allelic contrast (OR = 1.10; 95% CI = 0.76–1.60), dominant model (OR = 1.02; 95% CI = 0.81–1.27) and recessive model (OR = 0.87; 95% CI = 0.65–1.17) of 5HT2A C102T failed to show significant associations (Table 3, Figure 3). Furthermore, no significant association was identified from the pooled results when stratified by ethnicities and APOE ε4 (*p* > 0.05) (Table 3). In addition, we also conducted a subgroup analysis by classifying the Caucasian group into two subgroups (Italian and non-Italian) and obtained negative results (Italian: *p* = 0.34, OR = 0.86 95% CI = 0.64–1.17; non-Italian: *p* = 0.39, OR = 1.35 95% CI = 0.68–2.69).

**Table 3:** The association between 5HTTLPR and Alzheimer’s disease

SNPs (minor allele)	Genetic Model	Number of studies	Numbers		Test of association		Model	Test of heterogeneity	
			case	control	OR [95% CI]	*p*-Value		*P* value	I^2^ (%)
5HTTLPR (L)	Allelic(L)								
	total	16	4350	5764	1.10 [0.76, 1.60]	0.62	R	< 0.00001	94
	Asian	4	1048	1388	0.93 [0.66, 1.31]	0.67	R	0.06	60
	Caucasian	12	3302	4376	1.16 [0.72, 1.85]	0.54	R	< 0.00001	96
	With APOE ε4	5	628	330	1.31 [0.69, 2.48]	0.41	R	0.02	65
	Without APOE ε4	5	756	1188	1.50 [0.87, 2.59]	0.61	F	0.18	36
	Dominant(LL+LS/SS)								
	total	14	1884	2399	1.02 [0.81, 1.27]	0.88	R	0.01	53
	Asian	4	524	744	1.09 [0.84, 1.40]	0.52	F	0.92	0
	Caucasian	10	1360	1655	1.01 [0.73, 1.40]	0.96	R	0.002	66
	With APOE ε4	5	332	165	1.42 [0.86, 2.34]	0.17	F	0.36	9
	Without APOE ε4	5	378	594	0.94 [0.58, 1.52]	0.81	R	0.05	58
	Recessive(LL/LS+SS)								
	total	15	2098	2453	0.87 [0.65, 1.17]	0.36	R	< 0.0001	70
	Asian	4	524	744	1.10 [0.68, 1.78]	0.69	F	0.88	0
	Caucasian	11	1574	1709	0.83 [0.59, 1.17]	0.29	R	< 0.00001	78
	With APOE ε4	5	332	164	0.65 [0.36, 1.18]	0.16	F	0.78	0
	Without APOE ε4	5	378	594	0.91 [0.64, 1.30]	0.60	F	0.30	18

Abbreviations: 5HTTLPR: 5HTT gene-linked polymorphic region; L: long; S:short; APOE; Apolipoprotein E; R: random model; F: fixed model; OR: odds ratios; CIs: confidence intervals.

### Test of heterogeneity

Considering the great heterogeneity among studies, the random-effect model was applied. Heterogeneity was found for the 5HT2A allelic and recessive models of 5HT2A C102T and AD in Asian subgroup and subgroups without APOE ε4 (Table 2). The heterogeneity in this polymorphism was contributed mainly by Zhang et al. Removal of this study from meta-analysis gave 0% (*p* > 0.05) (Allelic contrast: Asian: *p* = 0.40; APOE ε4+: not available (NA); recessive model: Asian: NA; APOE ε4+: NA) heterogeneity and the result remained none significant, which showed that it had the highest effect on the correction of 5HT2A and AD. Furthermore, subgroup analysis stratified by ethnicities and APOE ε4 was performed and showed no obvious difference (Ethnicity: *p* = 0.1; APOE ε4: *p* = 0.55), implying that the ethnicity and APOE ε4 exerted no influence on the association between the 5HT2A C102T polymorphism and risk of AD.

Significant heterogeneities were also found in allelic, dominant, and recessive models of 5HTTLPR (L/S) (Table 3). The heterogeneity in this polymorphism was contributed mainly by Sukonick et al., Gru¨nblatt et al., and Tsai et al. Removal of these studies from meta-analysis gave 0–47% heterogeneities (*p* > 0.05). And subgroup analysis stratified by ethnicity and APOE ε4 was performed and showed no obvious difference (Ethnicity: *p* = 0.11; APOE ε4: *p* = 0.40), implying that the ethnicity and APOE ε4 exerted no influence on the association between the 5HTTLPR (L/S) polymorphism and risk of AD.

### Sensitivity analysis and publication bias

**Figure 4:**
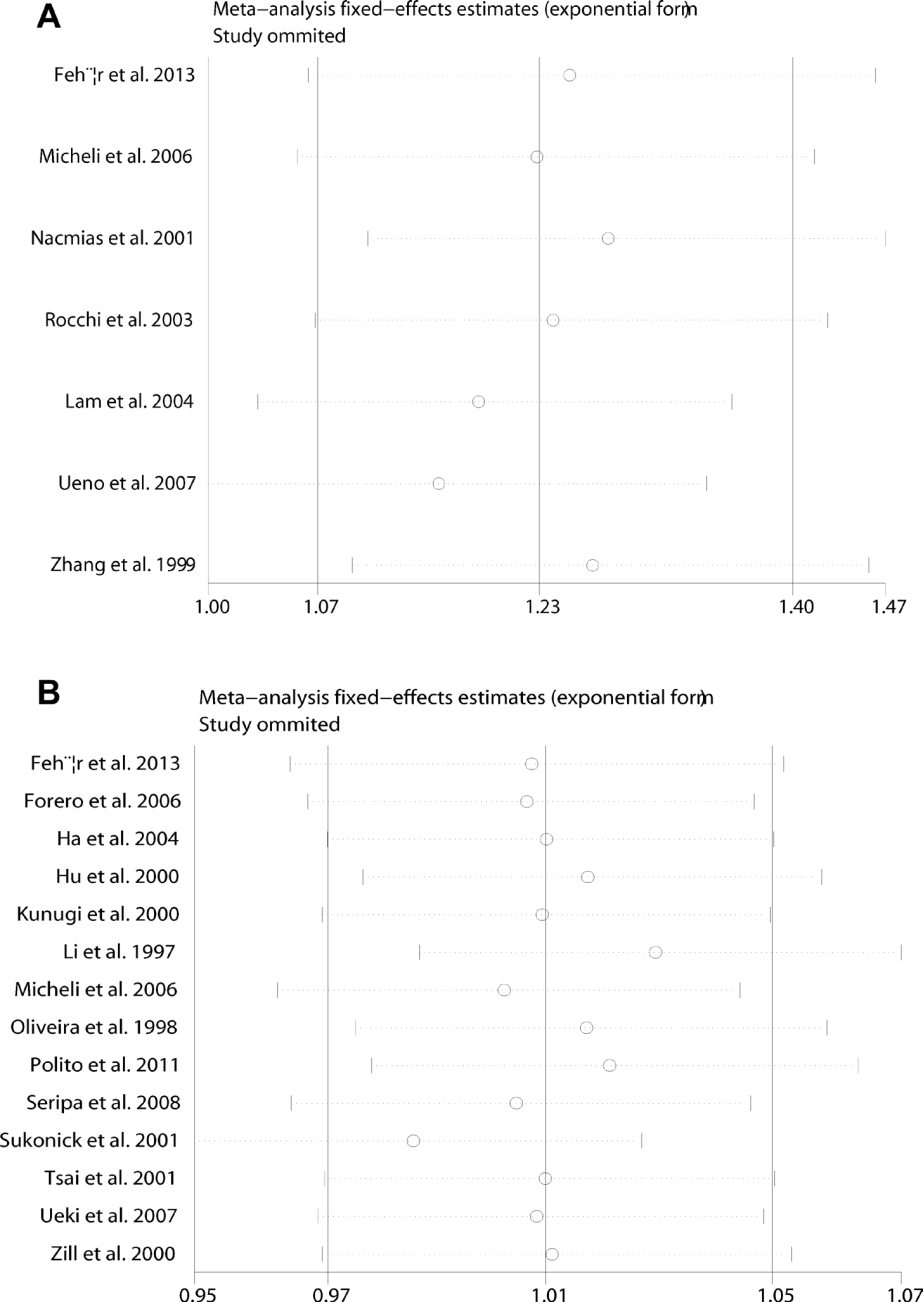
Sensitivity analyses between allelic models of 5HT2A C102T and 5HTTLPR (L/S) and AD (**A**) 5HT2A C102T; (**B**) 5HTTLPR (L/S).

**Figure 5:**
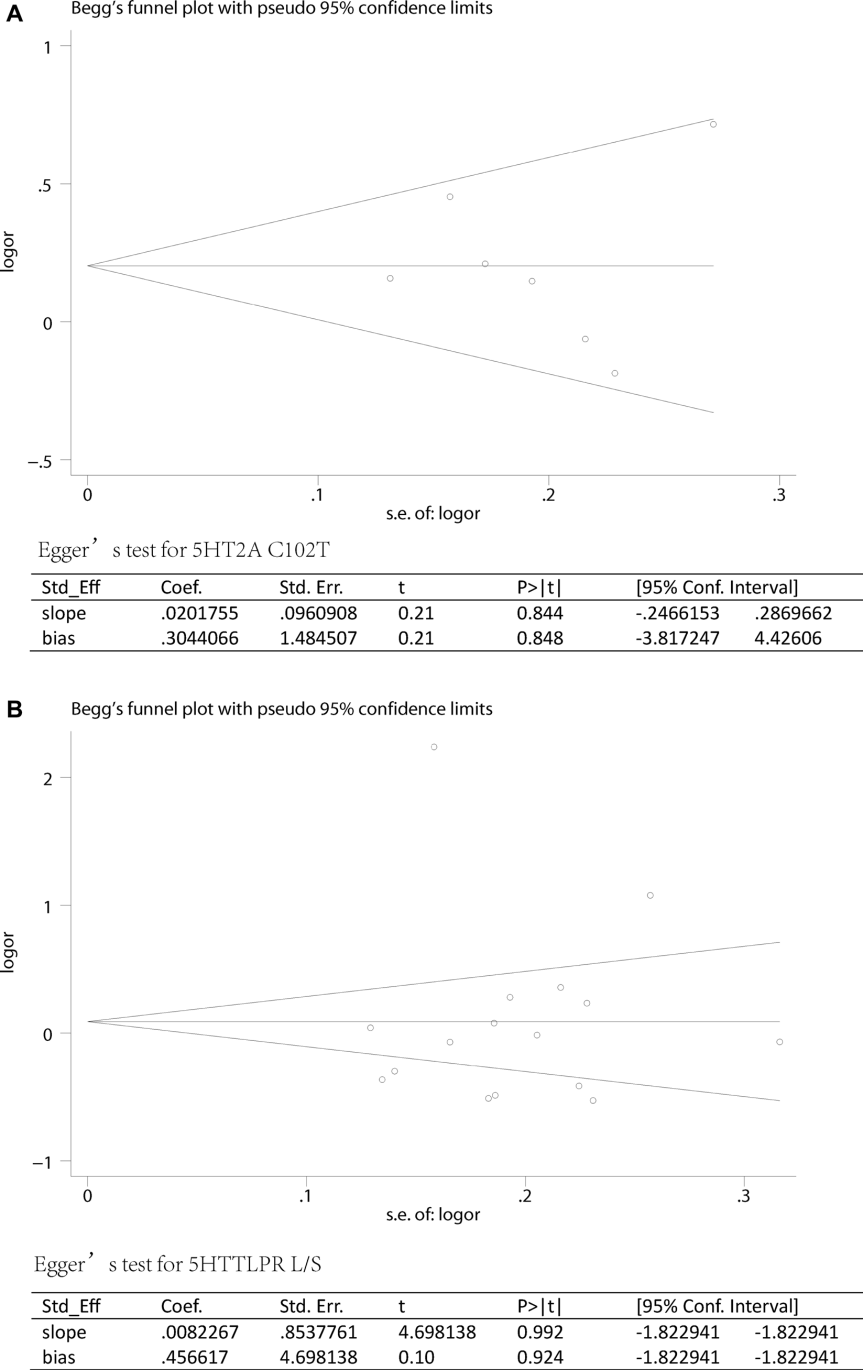
Publication biases of literatures for 5HT2A C102T and 5HTTLPR (L/S) were tested by Begg’s funnel plot and Egger’s test (**A**) 5HT2A C102T; (**B**) 5HTTLPR (L/S).

Sensitivity analysis which excluded the influence of a single study on the overall risk estimate by excluding one study at a time was confirmed. The ORs were not significantly altered in 5HT2A C and 5HTTLPR L (Figure 4). These evidences indicated that the present results were statistically stable and reliable. Funnel plots and Egger’s test were performed to assess publication bias. The results revealed that there was no obvious publication bias in overall analysis for 5HT2A C (p_egger_=0.955) and 5HTTLPR L (p_egger_=0.924) (Figure 5). The shape of Begg’s funnel plot did not reveal any obvious asymmetry (Figure 5),

## DISCUSSION

The combined results in this meta-analysis indicated that the allelic and dominant models of 5HT2A C102T were significantly associated with increased risk of AD among Asians and patients without APOE ε4. However, the present study failed to prove the hypothesis that the 5HTTLPR (L/S) was associated with AD.

The 5HT2A gene, which codes for the serotonin receptor type 2A, is located at 13q14-q21 [48]. The C/C genotype of 5HT2A C102T carriers showed a significant, 2-fold increased risk when compared to those who carrying the C or the T allele [49]. Increasing evidence revealed the 5HT2A C102T was a risk factor in many psychiatric diseases such as bipolar affective disorder, schizophrenia, AD, as well as BPSD in AD [50–52]. The results of the meta-analysis revealed significant associations between the allelic and dominant models of 5HT2A C102T and AD, However, the exact biological mechanism that the 5HT2A gene polymorphisms influence susceptibility to AD remains unclear. Subgroup analysis stratified by ethnicities revealed the AD risk increased significantly for allelic and dominant models of 5HT2A C102T in Asian population. Notable, three studies [25, 38–39] investigated the association between 5HT2A C102T and AD in Asian population. And two of them observed negative results. This discrepancy in individual studies and combined analysis may due to the limited sample size in individual studies. Furthermore, the subgroup analysis stratified by APOE ε4 in the present meta-analysis also indicated the allelic and dominant models of 5HT2A C102T increase the risk of AD in subgroup without APOE ε4. However, there were only two studies [38–39] included in subgroup analysis, which might cause insufficient power to detect slight association. To identify the 5HT2A C102T to be a specific risk factor for AD in Asian subjects and subgroup without APOE ε4, future larger-scale studies are necessary.

A functional polymorphism in the 5- regulatory promoter region, termed 5-HTTLPR, has been investigated in psychosis, mood disorder, BPSD, affective disorder, and AD [53–56]. 5-HTTLPR S allele leads to a decrease of 5-HTT mRNA transcription, 5-HTT ligand binding, and 5-HT uptake than 5-HTTLPR L allele [57]. However, the precise relationship between 5-HTTLPR (L/S) polymorphism and serotonin levels is still unclear. To date, a total of 16 studies have detected genetic association between 5-HTTLPR (L/S) and the risk of AD. And, 5 studies reported positive results [1, 31–33, 45]. We noticed that Polito et al. [33] has conducted a case-control and meta-analysis study with 13 individual studies and showed no significant association between the 5HTTLPR S allele and the risk of AD. Interestingly, we included 16 studies and reported negative results for the correction of allelic, dominant and recessive models of 5-HTTLPR (L/S) and AD as well. For the significant heterogeneity among studies, we introduced subgroup analysis by ethnicities and APOE ε4 and showed no association between 5-HTTLPR (L/S) and AD. We also investigated the association between 5-HTTLPR (L/S) and AD in Italian and non-Italian subgroups, and obtained similar results conducted by Polito et al [33]. All these negative results indicate the 5-HTTLPR (L/S) might not be the susceptible factor for AD.

Nonetheless, limitations also need to be acknowledged in our meta-analysis. Firstly, we enrolled a particularly small number of studies analyzing for association between the 5HT2A C102T and AD (7 case-control studies), which may result in an insufficient power for identifying relationship of 5HT2A C102T and AD risk. Secondly, we involved only Asian and Caucasian populations in the present study. Other populations such as African were not included. However, we could not assess the association in African population for lack of studies. Therefore, future studies on various ethnicities are needed. Thirdly, further subtle adjusted analysis by other co-variants such as ages, gender, education level, and life style should be carried out to obtain a more precise evaluation. Fourthly, AD was a progressive neurodegenerative disease with age and gender bias. It is necessary to analysis the genetic association between the 5HT2A (C102T) and 5HTTLPR (L/S) in subgroups stratified by age or gender.

In conclusion, our meta-analysis suggests that 5HT2A C102T may increase susceptibility to AD in Asian population and subgroup without APOE ε4 in both allelic and dominant models. And, the 5-HTTLPR (L/S) might not be the risk factor for AD. However, large-scale studies with more subjects are warranted to confirm these findings.

## MATERIALS AND METHODS

### Literature search strategy

This meta-analysis followed the Cochrane collaboration definition and PRISMA 2009 guidelines for meta-analysis and systematic review. Literatures search on PubMed, Embase, Web of Science, the Cochrane Library databases and Chinese National Knowledge Infrastructure (CNKI) was performed to investigate all relevant publications exploring the relationship between 5HT2A and 5HTTLPR polymorphisms and the risk of AD (up to June 1, 2017). The search terms were following: “5HT2A” or “neurotransmitter 5 hydroxytryptophan 2A Receptor” or “serotonin receptor 2A” or “serotonin 2A Receptor” or “HTR2A” and “polymorphism” or “variant” or “gene mutation” “single nucleotide polymorphism (SNP)” or “gene variation” and “Alzheimer’s disease” or “AD” and “promoter region of the serotonin transporter gene” or “5HTTLPR”. No language was limited. Meanwhile, other potentially relevant literatures were identified by cross-references within eligible studies.

### Inclusion/exclusion criteria

1) Investigating the association between 5HT2A (C102T), 5HTTLPR (L/S) polymorphisms and susceptible of AD. 2) The study was case-control and/or cohort designed. 3) Sufficient published data for calculating an odds ratio (OR) with 95% confidence interval (CI). 4) The genotype distributions in control groups were in the Hardy-Weinberg equilibrium (HWE).

### Exclusion criteria

1) Duplicated studies, abstracts, letters or reviews. 2) Studies without controls. 3) Control group did not confirm to Hardy-Weinberg equilibrium (HWE). 4) No available genotype data.

### Data extraction and quality assessment

Data abstraction was performed independently by L. T. and Y. W. The following information from each study was summarized: first author, year, ethnicity, numbers of cases and controls, mean age and gender, methods of genotyping, sample source, Hardy-Weinberg equilibrium (HWE) for control groups. All included studies were evaluated using the Newcastle-Ottawa Scale (NOS) independently by L. T. and Y. W. C. Any discrepancies in the assessment were resolved by J. M. L.

### Statistical analyses

The odds ratio (OR) and 95% confidence interval (95% CI) were calculated for evaluating the association between 5HT2A T102C, 5HTTLPR L/S and AD using the RevMan 5 (Oxford, UK) and STATA12.0 (StataCorp, College Station, TX, USA). The pooled ORs were calculated in the allelic, dominant and recessive models. The statistical significance of the OR was determined using the Z test. Statistical heterogeneity was tested using χ^2^-based Q test and the *I*^2^ statistic. When there was no significant heterogeneity across studies (*I*^2^ < 50%), the fixed effect model (Mantel-Haenszel method) was used for meta-analysis. Otherwise, the random effect model (the DerSimonian and Laird method) was used. Sources of heterogeneity were evaluated by stratification analysis of ethnicities and APOE ε4 allele, according to the study characteristics. Sensitivity analysis was performed to assess the stability of results. The publication bias was detected with Begg’s test and Egger’s test. *p* < 0.05 was considered statistically significant.

## SUPPLEMENTARY MATERIALS TABLES


