# Supplementary material for: The association of 5HT2A and 5HTTLPR polymorphisms with Alzheimer’s disease susceptibility: a meta-analysis with 6945 subjects
Source: Oncotarget. 2017 Dec 22;9(19):15077–89. doi: 10.18632/oncotarget.23611 (PMC5871099; doi:10.18632/oncotarget.23611)
Supplement: Supplementary file 1 [file oncotarget-09-15077-s001.pdf]

The association of 5HT2A and 5HTTLPR polymorphisms with Alzheimer's disease susceptibility: a meta-analysis with 6945 subjects

SUPPLEMENTARY MATERIALS

Supplementary Table 1: Methodological quality of the included studies according to the Newcastle-Ottawa scale

Variants	Author (Year)	Ethnicity	Adequacy of Case Definition	Representativeness of the Cases	Selection of Controls	Definition of Controls	Comparability Cases/Controls	Ascertainment of Exposure	Same Method of Ascertainment	Non-response rate	Total
5HT2A C102T	Lam et al. 2004	Chinese	*	*	NA	NA	**	*	*	*	7
	Rocchi et al. 2003	Itailan	*	*	*	*	**	*	*	*	9
	Micheli et al. 2006	Itailan	*	*	*	*	**	*	*	*	9
	Nacmias et al. 2001	Itailan	*	*	*	NA	**	*	*	*	8
	Ueno et al. 2007	Japanese	*	*	*	*	**	*	*	*	9
	Zhang et al. 1999	Chinese	*	*	NA	NA	**	*	*	*	7
	Fehér et al.2013	Hungarian	*	*	*	*	**	*	*	*	9
5HTTLPR L/S	Kunugi et al. 2000	Japanese	*	*	NA	NA	**	*	*	*	7
	Ha et al 2004	Korean	*	*	*	*	**	*	*	*	9
	Tsai et al.2001	Chinese	*	*	*	*	**	*	*	*	9
	Ueki et al. 2007	Japanese	*	*	*	*	**	*	*	*	9
	Fehér et al.2013	Hungarian	*	*	*	*	**	*	*	*	9
	Lorenzi et al. 2010	Itailan	*	*	*	*	**	*	*	*	9
	Forero et al. 2006	Colombian	*	*	NA	NA	**	*	*	*	7
	Gru¨nblatt et al. 2009	Austrian	*	*	*	*	**	*	*	*	9
	Hu et al. 2000	Germany	*	*	NA	NA	**	*	*	*	7
	Li et al. 1997	British	*	*	*	NA	**	*	*	*	8
	Oliveira et al. 1998	Brazil	*	*	NA	NA	**	*	*	*	7
	Polito et al. 2011	Itailan	*	*	*	*	**	*	*	*	9
	Seripa et al. 2008	Itailan	*	*	*	*	**	*	*	*	9
	Sukonick et al. 2001	American	*	*	*	*	**	*	*	*	9
	Zill et al. 2000	Germany	*	*	*	NA	**	*	*	*	8

This table identifies 'high' quality choices with a 'star'. A study can be awarded a maximum of 1 star for each numbered item within the Selection and Exposure categories. A maximum of 2 stars can be given for Comparability. *, Yes; NA, not applicable.

Supplementary Table 2: The association between 5HTTLPR and Alzheimer's disease stratified by Italian and nonItalian

SNPs (minor allele)	Genetic Model	Number of studies	Numbers		Test of association		Model	Test of heterogeneity	
			case	control	OR [95% CI]	*p*-Value		*P* value	I^2 (%)
5HTTLPR (L)	Allelic(L)								
	Caucasian	12	3302	4376	1.16 [0.72, 1.85]	0.54	R	< 0.00001	96
	Italian	4	1394	962	0.86 [0.64, 1.17]	0.34	R	0.03	67
	Non-Italian	8	1908	3414	1.35 [0.68, 2.69]	0.39	R	< 0.00001	97
	Dominant(LL+LS/SS)								
	Caucasian	10	1360	1655	1.01 [0.73, 1.40]	0.96	R	0.002	66
	Italian	7	827	1228	0.98 [0.61, 1.57]	0.94	R	0.001	73
	Non-Italian	3	421	332	1.11 [0.73, 1.69]	0.63	F	0.17	44
	Recessive(LL/LS+SS)								
	Caucasian	11	1574	1709	0.83 [0.59, 1.17]	0.29	R	< 0.00001	78
	Italian	4	697	481	0.87 [0.51, 1.47]	0.59	R	0.007	76
	Non-Italian	7	877	1228	0.81 [0.50, 1.33]	0.41	R	< 0.0001	81

Abbreviations: 5HTTLPR: 5HTT gene-linked polymorphic region; L: long; S:short; R: random model; F: fixed model; OR: odds ratios; CIs: confidence intervals.
